# The Alpha-Melanocyte Stimulating Hormone Induces Conversion of Effector T Cells into Treg Cells

**DOI:** 10.1155/2011/246856

**Published:** 2011-09-15

**Authors:** Andrew W. Taylor, Darren J. Lee

**Affiliations:** Department of Ophthalmology, Boston University School of Medicine, 72 East Concord Street, Boston, MA 02118, USA

## Abstract

The neuropeptide alpha-melanocyte stimulating hormone (*α*-MSH) has an important role in modulating immunity and homeostasis. The production of IFN-*γ* by effector T cells is suppressed by *α*-MSH, while TGF-*β* production is promoted in the same cells. Such *α*-MSH-treated T cells have immune regulatory activity and suppress hypersensitivity, autoimmune diseases, and graft rejection. Previous characterizations of the *α*-MSH-induced Treg cells showed that the cells are CD4^+^ T cells expressing the same levels of CD25 as effector T cells. Therefore, we further analyzed the *α*-MSH-induced Treg cells for expression of effector and regulatory T-cell markers. Also, we examined the potential for *α*-MSH-induced Treg cells to be from the effector T-cell population. We found that the *α*-MSH-induced Treg cells are CD25^+^  CD4^+^ T cells that share similar surface markers as effector T cells, except that they express on their surface LAP. Also, the *α*-MSH treatment augments FoxP3 message in the effector T cells, and *α*-MSH induction of regulatory activity was limited to the effector CD25^+^ T-cell population. Therefore, *α*-MSH converts effector T cells into Treg cells, which suppress immunity targeting specific antigens and tissues.

## 1. Introduction

The melanocortin pathway is a highly conserved family of receptors and ligands that have important roles in regulating metabolism, pigmentation, and immunity [1–3]. The prototypic melanocortin, alpha-melanocyte stimulating factor (*α*-MSH), suppresses inflammation mediated by both innate and adaptive immunity. The neuropeptide *α*-MSH inhibits NF-kB activation by blocking the intracellular signaling pathways initiated by TLR, scavenging, IL-1, and TNF*α* receptors in macrophages, dendritic cells, and neutrophils through the melanocortin 1 receptor (MC1r) and the melanocortin 3 receptor (MC3r) [4–11]. In addition, *α*-MSH induces MC1r expression, and transcription of proopiomelanocortin hormone in monocytes establishing a self-perpetuating immunosuppressive autocrine loop [12]. Because the serum levels of *α*-MSH increase during the acute phase of inflammation [13] and the anti-inflammatory activity of *α*-MSH has an antagonist relationship with proinflammatory mediators [2] suggests that *α*-MSH has an important role in the resolution of inflammation and the maintenance of immune homeostasis. 

The analysis of the molecular mechanisms of ocular immune privilege demonstrates the importance of *α*-MSH in preventing and suppressing inflammation within the healthy eye [14, 15]. Within the ocular microenvironment, the constitutively present *α*-MSH has an important role in suppressing the activation of effector T-cells, and in the regional induction of Treg cells [14, 16–19]. Also, the melanocortin pathway has a role in resolving ocular autoimmune disease in converting a systemic effector T cell response into a regulatory T-cell response specific to ocular autoantigens [20, 21]. Injections of *α*-MSH peptide or DNA plasmids encoding for *α*-MSH have been used as experimental therapy to suppress graft rejection, inflammation, and autoimmune diseases in the eye and in the CNS [22–28]. Also, Treg cells induced by *α*-MSH have been used in adoptive transfer experiments to suppress hypersensitivity, autoimmunity, and graft rejection [17, 29, 30]. These adoptive transfer experiments demonstrated that the activation of regulatory activity was antigen specific; however, the mechanisms of suppression were general and most likely mediated by TGF-*β* produced by the Treg cells [17]. 

 We have previously demonstrated that *α*-MSH not only suppresses IFN-*γ* production by activated effector T cells already programmed to be Th1 cells, but also made the T cells become functional CD25^+^ CD4^+^ Treg cells that produce only TGF-*β* [16, 17, 29]. To see the effects of *α*-MSH on the effector T cells; it is required for the effector T cells to be stimulated through their T-cell receptor (Tcr)-stimulation either by antigen presenting cells or by cross-linking using anti-CD3 antibody 2C11. The effects of *α*-MSH is not through the Tcr, but through the melanocortin 5 receptor (MC5r) [17]. Blocking MC5r prevents *α*-MSH induction of regulatory activity in the activated T cells. Further analysis of the *α*-MSH-induced Treg cells show that the Treg cells after activation express message for IFN-*γ* and TGF-*β* at the same level as untreated activated Th1 cells [16]. Staining for intracellular IFN-*γ* protein shows similar levels of the protein in *α*-MSH-treated and -untreated effector T cells [31]. Treatment with *α*-MSH enhances ubiquitination of intracellular IFN-*γ* protein preventing the T cells from secreting IFN-*γ* while allowing for free production and secretion of TGF-*β*. This demonstrated that *α*-MSH manipulates effector CD4^+^ T cells as they are Tcr-stimulated by altering the secretion of specific cytokines to make the T cells suppress or regulate immunogenic inflammation. This is not a transient effect, since it is possible to adoptively transfer these *α*-MSH-induced Treg cells and effectively suppress hypersensitivity, autoimmune disease, and graft rejection in vivo [17, 29, 30].

In this work, we assayed the *α*-MSH-induced Treg cells and found that they are CD25^+^ CD4^+^ T cells, producing TGF-*β* and expressing on their surface latency-associated peptide of TGF-*β* (LAP). In addition, the results further support the potential for *α*-MSH to convert effector T cells into functional Treg cells.

## 2. Materials and Methods

### 2.1. In Vitro Simulation and *α*-MSH Treatment of Effector T Cells

C57BL/6 mice were purchased from Jackson Laboratories (Bar Harbor, ME). All mice were treated with the approval of the Schepens Eye Research Institute and the Boston University School of Medicine Institutional Animal Care and Use Committees. The immunization of the mice and isolation of lymph node T cells were as we have done before [14, 17, 30, 31]. The mice were injected with 50 *μ*L of Complete Freund's Adjuvant fortified with 10 mg/mL desiccated *Mycobacterium tuberculosis* into the footpad. Seven days later; the draining popliteal lymph node was collected to obtain effector T cells. The lymph nodes were removed and placed in 5% fetal bovine serum (FBS) in RPMI-1640 supplemented with 10 *μ*g/mL Gentamycin (Sigma, St Louis, MO), 10 mM HEPES, 1 mM Sodium Pyruvate (BioWhittaker, Walkersville, MD), and 1X Nonessential Amino Acids (NEAA). The lymph nodes were made into a single cell suspension, depleted of red blood cells, and washed with serum free media (SFM), RPMI-1640 supplemented with 0.2% ITS+1-media supplement (Sigma), and 0.1% bovine serum albumin (BSA). The CD4^+^ T cells were isolated from the cell suspension using negative selection CD4 columns (R&D Systems, Minneapolis, MN). The isolated CD4^+^ T cells (98% CD4^+^ by flow cytometry analysis) were plated into the wells of 96-well plate at 1 × 10^6^ cells per well. Into each well was added 1 *μ*g anti-CD3 (Tcr) 2C11 antibody (BD Biosciences, San Diego, CA), and *α*-MSH (Bachem, Torrance, CA) at a physiological concentration of 30 pg/mL [14]. The cultures were incubated at 37°C, 5% CO_2_ until collected for the assays described below.

### 2.2. Flow Cytometry Staining

Antibodies used for flow cytometry staining were anti-CD25-PerCP-Cy5.5 (BD Biosciences), anti-GITR-FITC (R&D Systems), anti-CTLA4-FITC (R&D Systems), anti-CD127-PE (BD Biosciences), CD44-FITC (BD Biosciences), CD62L-FITC (BD Biosciences), anti-LAP-PerCP (R&D Systems), anti-CD4-AF700 (Biolegend, San Diego, CA), and anti-CD25-APC-Cy7 (Biolegend). The cultured cells were collected at 72 hours or at 48 hours for LAP staining, and washed once in ice cold staining buffer (0.01 M PBS with 1% BSA). The cells were resuspend in staining buffer, and all the cells were stained for CD25. The cells were costained for GITR, CTLA-4, CD44, CD62L, or LAP. The cells were incubated with the antibodies for 30 minutes on ice, washed twice with ice cold staining buffer, and resuspended in staining buffer. The cells were filtered through nylon mesh and analyzed by flow cytometry. The flow cytometry data was evaluated using FlowJo software (Tree Star, Inc, Ashland, OR) gating on the CD25^+^ T cells. All flow cytometry results presented are representative of two independent experiments.

### 2.3. Quantitative Real-Time PCR for FoxP3

Total RNA was isolated from 4- and 24-hour-cultured T cells with RNeasy mini kit (Qiagen Inc.,Valencia, CA) according to the manufacturer's instructions. The cells were washed three times with PBS buffer and collected. They were disrupted, lysed, and homogenized using a QIAshredder spin column (Qiagen Inc.). An equal volume of 70% ethanol was mixed with the homogenized lysates and applied to RNeasy mini column for adsorption of total RNA to column membrane. RNA was washed once with washing buffer. Further DNA removal was done using a column DNase digestion by RNase-Free DNase Set (Qiagen Inc.). RNA was washed and eluted in RNase-free water. The RNA concentration was determined by spectrophotometry at 260 nm.

The first-strand cDNA synthesis reaction was undertaken with SuperScript First-Strand Synthesis System for RT-PCR kit (Invitrogen, Carlsbad, CA). The isolated total RNA (5 *μ*g) was reverse transcribed in a 20 *μ*L reaction mixture containing 20 mM Tris-HCl (pH 8.4), 50 mM KCl, 200 *μ*M of each dNTP, 50 ng of random hexamers, 5 mM MgCl_2 _10 mM DTT, 40 units of RNaseOUT Recombinant Ribonuclease Inhibitor, and 50 units of SuperScript II RT. The mixture was incubated at 25°C for 10 min and transferred to 42°C for 50 min. The RT reaction was terminated by heating the mixture to 70°C for 15 min and then adding 2 units of RNase H at 37°C for 20 min to remove the RNA from the cDNA:RNA hybrid molecule.

The 50 *μ*L of real-time PCR reaction mixture consisted of a FAM-dye labeled TaqMan MGB predesigned mouse FoxP3 probe of two unlabeled PCR primers, FoxP3F (5′ CAGCTGCCTACAGTGCCCCTAG) and FoxP3R (5′ CATTTGCCAGCAGTGGGTAG), (Applied Biosystems, Foster City, CA), 25 *μ*L of 2X TaqMan Universal PCR Master Mix containing AmpliTaq Gold DNA Polymerase, AmpErase UNG, dNTPs with dUTP (Applied Biosystems), and 2 *μ*L of our sample cDNA (equivalent to 500 ng of RNA). The final reaction concentration of the probes was 250 nM and 900 nM for each primer. Amplification and detection of PCR products were performed using an ABI Prism 7900HT Sequence Detection System with thermal cycling conditions of: 2 min at 50°C for 1 cycle, 10 min at 95°C for 1 cycle, 15 sec at 95°C, and 1 min at 60°C for 40 cycles. The results were analyzed with SDS 2.1 software (Applied Biosystems). Each assay was carried out in triplicate. The relative expression of FoxP3 mRNA was normalized to the relative expression of GAPDH mRNA (Applied Biosystems). The relative quantization of the FoxP3 mRNA in each sample was measured using the comparative Ct (threshold cycle) method.

### 2.4. Sorting, Cytokine Production, and Regulatory T-Cell Assay of *α*-MSH-Treated CD25^−^ T Cells

Purified CD4^+^ T cells were stained with anti-CD25-FITC (BD Biosciences) and sorted on the Cytomation MoFlo Cell Sorter (Beckman Coulter, Brea, CA). The CD25^−^ T cells were collected, and cultured at 4 × 10^5^ cells per well of a 96 well plate. To the cell cultures were added 1 *μ*g of anti-Tcr antibody, and 30 pg/mL of *α*-MSH. After 48 hours, the supernatants were collected and IFN-*γ* and TGF-*β* were measured. The IFN-*γ* concentration was measured by ELISA using the R&D System dual antibody kit. The TGF-*β* concentration was measured using the standard MV1Lu bioassay [18]. To assay for regulatory activity by the *α*-MSH-treated CD25^−^ T cells, the T cells were collected from the cell cultures and 2 × 10^5^ *α*-MSH-treated CD25^−^ T cells were added to cultures with 2 × 10^5^ anti-Tcr-activated Th1 cells. After 48 hours of incubation, IFN-*γ* production was measured by ELISA. 

## 3. Results

### 3.1. Surface Marker Expression by *α*-MSH-Induced Treg Cells

**Figure 1:**
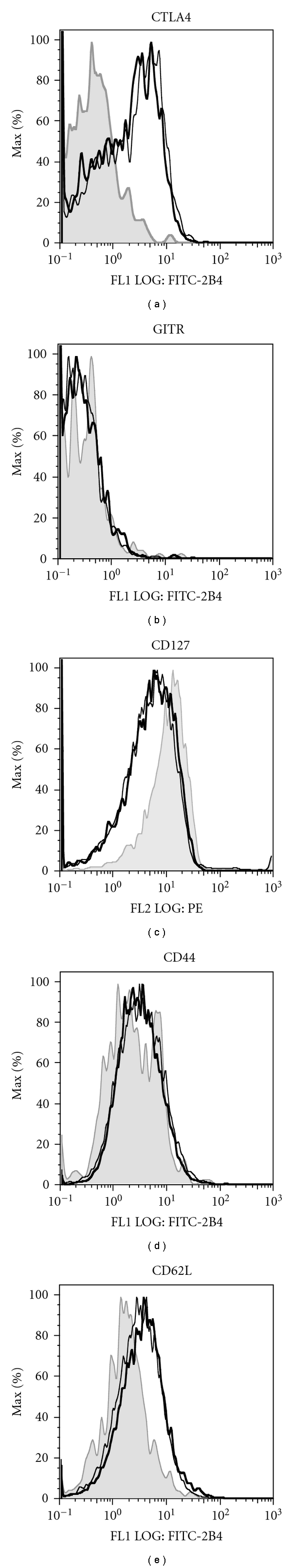
Flow cytometry analysis of effector/memory T-cell markers on *α*-MSH-induced Treg cells. Isolated draining lymph node CD4 T cells from immunized mice were stimulated and treated with *α*-MSH (30 pg/mL). After 72 hours of incubation, all the cells were stained for CD25. The cells were also stained for CTLA4, GITR, CD127, CD44, or CD62L. The costained cells were analyzed by flow cytometry. Presented results are a representative of two experiments showing the histogram expression of CTLA4, GITR, CD127, CD44, or CD62L on the gated CD25^+^ T-cell population. The gray shaded histograms are resting T cells; the thin lines are effector T cells; the thick lines are the *α*-MSH-induced Treg cells. There was no significant difference in the expression of CTLA4, GITR, CD127, CD44, or CD62L on the *α*-MSH-induced Treg cells compared to the stimulated effector T cells.

The *α*-MSH-induced Treg cells are CD25^+^ CD4^+^ T cells previously demonstrated to be a stable population of T cells that suppress the activation of inflammation mediated by effector T cells [16, 17, 28–30]. While it is considered that CD25 is a marker of Treg cells, *α*-MSH-induced Treg cells do not express CD25 any more than effector T cells [17]. Therefore, *α*-MSH-induced Treg cells were assayed and compared to effector T cells and resting T cells for CTLA4, GITR, CD127, LAP, CD44, and CD62L, which are other Treg and effector T-cell activation markers. The *α*-MSH-induced Treg cells were generated in vitro as done before by treating Tcr-stimulated CD4^+^ effector T cells with *α*-MSH at a physiological concentration of 30 pg/mL. After 72 hours, the cells were stained and assayed by flow cytometry gating on the CD25^+^ T cells (Figure 1). There was no difference found in the expression of CTLA4, GITR, CD127, CD44, and CD62L between the *α*-MSH induced Treg cells and effector T cells. The effector T cells and the *α*-MSH-induced Treg cells expressed CTLA4, CD127, CD44, and CD62L but not GITR. The cells had increased expression of CTLA4 and CD62L with depressed expression of CD127 compared to resting T cells. These results show that *α*-MSH-induced Treg cells express markers associated with effector T cells and not necessarily markers of Treg cells. In addition, the regulatory and effector CD25^+^ CD4^+^ T cells expressed both CD44 and CD62L suggesting that we may have induced T cells that could also function as memory T cells.

**Figure 2:**
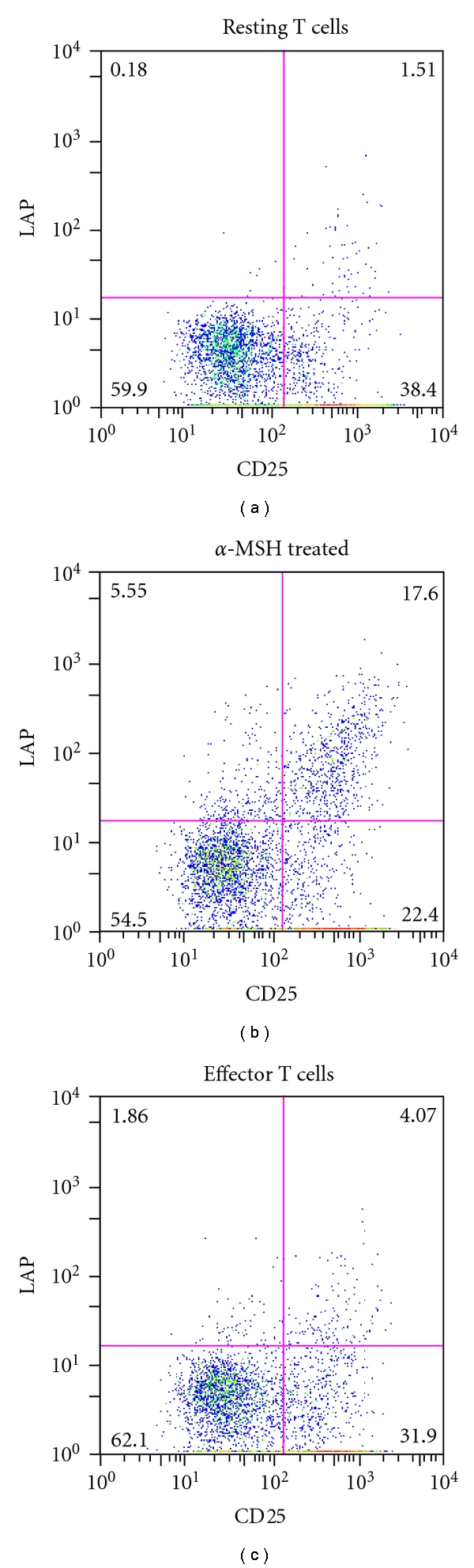
Flow cytometry analysis of LAP expression on *α*-MSH-induced Treg cells. The T cells were collected and treated as in Figure 1, but were costained for CD25 and LAP. The flow cytometry results are presented as dot plots showing T cells expressing CD25 and LAP representing the results of two independent experiments. The expression of LAP on the effector T cells decreases relative to the resting T cells. In contrast, there is more than a 10-fold increase in LAP expression on *α*-MSH-induced Treg cells compared to the effector T cells.

Previously reported findings demonstrated that the suppressive activity of *α*-MSH-induced Treg cells is mediated through TGF-*β* [17]. Flow cytometry analysis for LAP showed significant differences in the surface expression of LAP on the *α*-MSH-induced CD25^+^ CD4^+^ Treg cells to the CD25^+^ CD4^+^ effector T cells. The coexpression of CD25 and LAP shows that the *α*-MSH-induced CD25^+^ CD4^+^ Treg cells are highly expressive of LAP on their cell surface (Figure 2). There was greater than 10-fold increase in LAP expression on the *α*-MSH-induced Treg cells compared to the stimulated effector CD25^+^ CD4^+^ T cells. There is just as striking an increase in LAP expression on *α*-MSH-treated CD25^−^ CD4^+^ T cells, suggesting that *α*-MSH has a general effect of increasing the number of T cells expressing LAP. This could be due to increased availability of LAP for the T cells to bind or to general induction of TGF-*β* even in CD25^−^ T cells. Therefore, while the *α*-MSH-induced Treg cells express effector and possible memory T-cell markers, they differ from effector T cells by their surface expression of LAP.

### 3.2. FoxP3 Expression in *α*-MSH Induced Treg Cells

**Figure 3:**
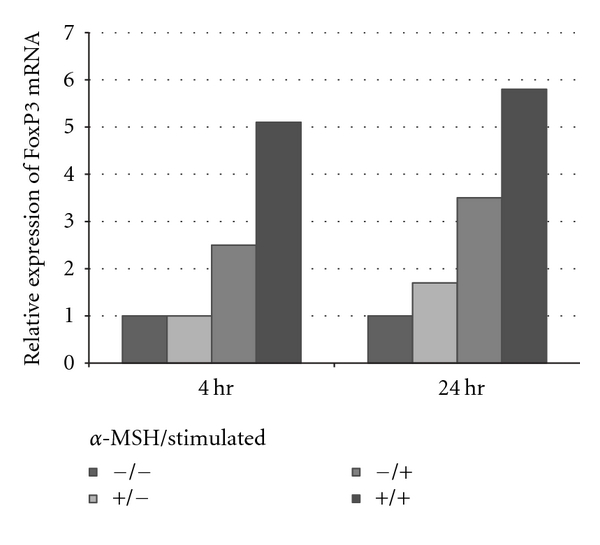
Real-time PCR analysis of FoxP3 after *α*-MSH treatment. The CD4^+^ T cells were isolated from draining lymph nodes, stimulated and treated with *α*-MSH as before. At 4 hours and 24 hours after treatment, message was isolated and analyzed by RT-PCR. Presented results are representative of two independent experiments showing the relative levels of FoxP3 message in CD4^+^ T cells. There is consistently about a 2 fold-higher amount of FoxP3 message in the *α*-MSH-treated (+/+) than in the untreated effector T cells (−/+).

The *α*-MSH-induced Treg cells were assayed by RT-PCR for expression of FoxP3 message at 4 and 24 hours after treatment (Figure 3). The *α*-MSH treatment induces a substantial early 2-fold upregulation in FoxP3 message in the activated T cells that is relatively maintained 24 hours later. This suggests that part of the mechanisms of *α*-MSH induction of regulatory activity involves FoxP3. The untreated effector T cells showed some increase in FoxP3 message that was less than the *α*-MSH-treated cells but greater than unstimulated effector T cells (resting T cells). This suggests that, although *α*-MSH treatment augments FoxP3 expression, there is naturally in the untreated effector T-cell population inducible FoxP3 expressing T cells.

### 3.3. The Effects of *α*-MSH on CD25^+^ or CD25^−^ T Cells

**Figure 4:**
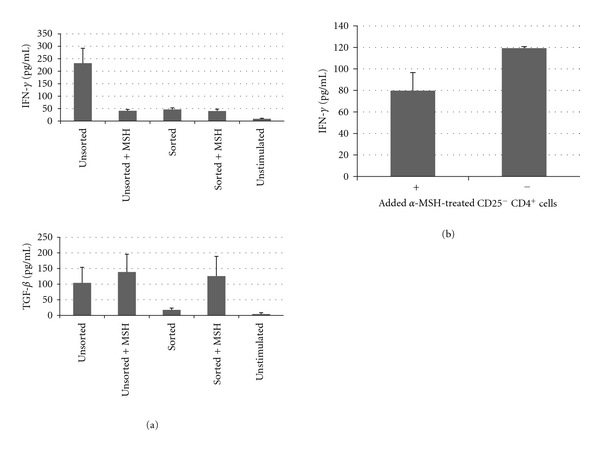
The effects of *α*-MSH on CD25^−^ CD4^+^ T cells. The CD4 T cells were isolated from the draining lymph node as in Figure 1 and were stained with CD25 antibody. The stained cells were sorted, and the CD25^−^ cells were placed in culture, stimulated, and treated with *α*-MSH. (a) After 48 hours, the supernatant was assayed for IFN-*γ* and TGF-*β*. The sorted CD25^−^ CD4^+^ T cells (Sorted) did not produce IFN-*γ* or TGF-*β*; however, when treated with *α*-MSH (Sorted + MSH) did produce TGF-*β* but not IFN-*γ*. (b) The *α*-MSH-treated CD25^−^ CD4^+^ T cells were transferred to cultures of activated Th1 cells. After 48 hours of incubation IFN-*γ* was measured in the supernatant. There was no statistical difference in IFN-*γ* production by the Th1 cells in culture with or without *α*-MSH-treated CD25^−^ CD4^+^ T cells.

To demonstrate that *α*-MSH treatment targets the activity of activated effector T cells, the effector T cells (CD25^+^ T cells) were removed from the collected draining lymph node CD4^+^ T cells before *α*-MSH treatment. The unsorted population of effector T cells produced IFN-*γ* and TGF-*β* and when treated with *α*-MSH produced only TGF-*β* (Figure 4(a)) similar to the cytokine production we have demonstrated before between effector T cells and *α*-MSH-induced Treg cells [14, 16, 17, 24, 28, 30]. The collected CD4^+^ T cells depleted of CD25^+^ T cells were completely devoid of all effector T-cell activity with no IFN-*γ* or TGF-*β* produced (Figure 4(a)). Treating these CD25^−^ CD4^+^ T cells with *α*-MSH-induced TGF-*β* production. This corresponds with the increase of LAP surface expression on CD25^−^ T cells treated with *α*-MSH seen in Figure 2. There was no expression of CD25 on the *α*-MSH-treated and *α*-MSH-untreated sorted T cells (data not shown). The *α*-MSH-treated CD25^−^ T cells did not suppress IFN-*γ* production by other Th1 cells in culture (Figure 4(b)). Therefore, independent of *α*-MSH induction of regulatory activity, *α*-MSH mediates TGF-*β* production, and the *α*-MSH-induced Treg cells come from *α*-MSH mediating regulatory activity in effector T cells.

## 4. Discussion

The results in this paper propose that *α*-MSH induction of regulatory immunity is mediated by *α*-MSH converting effector T cells. The *α*-MSH-induced Treg cells are CD25^+^ CD4^+^ expressing CTLA4, CD44, CD62L, and LAP. The effector CD25^+^ CD4^+^ T cells expressed the same markers except for LAP. While *α*-MSH-induced TGF-*β* production, it could not induce regulatory activity in naive T cells. This limitation of *α*-MSH induction of regulatory activity in effector T cells and not in naive T cells has been observed by us many times before but unreported. This implies that the direct effects of *α*-MSH on T cells are limited to antigen-experienced effector T cells. This explains why it is possible to use *α*-MSH to induce antigen-specific Treg cells that target antigen-driven autoimmunity, hypersensitivity, and graft rejection [16, 17, 29, 30]. Such an effect of *α*-MSH on effector T cells has implications not only on our own interests in ocular immune privilege but also on the role of *α*-MSH in immune homeostasis.

Since it has been speculated that one of the systemic roles of *α*-MSH is to promote resolution of inflammation [2], the ability of *α*-MSH to induce regulatory activity in activated effector T cells would contribute to resolving inflammation. The suppression of inflammation by *α*-MSH is a balance between its antagonistic activity with proinflammatory cytokines and activators of innate immunity. Therefore, during the resolution phase as proinflammatory signals decline, or are no longer present, the ability for *α*-MSH to induce regulatory activity increases. Because we have found that there is very little difference between effector T cells and the *α*-MSH-induced Treg cells except for their secreted cytokine profiles and surface expression of LAP, it is to be seen if the maintenance and activation of *α*-MSH-induced Treg cells could also be in balance with proinflammatory signals that drive adaptive immunity.

The constitutive presence of *α*-MSH within the ocular microenvironment contributes to the immunosuppressive mechanisms of ocular immune privilege [14]. The neuropeptide is part of the active ocular mechanisms that suppress effector T-cell activation [32]. Moreover, the presence of *α*-MSH is part of the mechanisms used by the ocular microenvironment to turn immunity onto itself, should effector cells be activated within the ocular microenvironment [33]. Recently found are the presence of autoantigen-specific Treg cells within the spleens of mice that have naturally recovered from ocular autoimmune disease [21]. Their presence in the spleen is dependent on the expression of MC5r, the receptor on T cells through which *α*-MSH induces regulatory activity [20]. In addition, *α*-MSH treatment of mice with experimental autoimmune encephalomyelitis (EAE) not only prevented further paralysis but also promoted an early recovery of full-motor function [24]. Moreover, in the spleens of these *α*-MSH-treated mice were Treg cells that suppress EAE. Therefore, augmenting *α*-MSH has the potential to promote activation of regulatory activity in effector T cells during an inflammatory response. Also, it appears that once the T cells are set in this direction, it may be difficult for *α*-MSH-induced Treg cells to revert back into effector T cells.

Very little is understood about the intracellular signals triggered by *α*-MSH within T cells. The melanocortin receptors are G-coupled protein receptors that elevate cAMP when bound with *α*-MSH [34]; however, this alone cannot account for all the actions induced by *α*-MSH in T cells. The induction of regulatory activity by *α*-MSH in T cells is through MC5r [17]. When *α*-MSH binds MC5r it has been found to activate within immune cells the JAK2/STAT1 and ERK1/2 pathways [35, 36]. Therefore, *α*-MSH through MC5r is at least potentially linked to signals that mediate cellular differentiation and cytokine production. What is not known is how this is linked to *α*-MSH-mediated induction of Treg cells. 

The *α*-MSH-induced Treg cells are T cells that retain several features of effector T cells and have also features and functions associated with natural and inducible Treg cells. Previous publications have shown that the *α*-MSH-induced Treg cells are limited in cytokine production to TGF-*β*, proliferate, must be activated through their Tcr to mediate regulatory activity, and their induction is dependent on the expression of MC5r [17]. To these findings the *α*-MSH-induced Treg cells express surface LAP, corresponding to their use of TGF-*β* to suppress effector T cells through contact or enhanced release of active TGF-*β* [17]. Also, the *α*-MSH-induced Treg cells are from the effector CD25^+^ CD4^+^ T-cell population indicating *α*-MSH-mediated conversion of effector T cells into Treg cells.
